# Pre-Degassed Microfluidic Chamber-Based Digital PCR Device for Meat Authentication Applications

**DOI:** 10.3390/mi12060694

**Published:** 2021-06-14

**Authors:** Hezhi Hu, Jingmeng Cheng, Chunyang Wei, Shanshan Li, Chengzhuang Yu, Xiaoshuai Meng, Junwei Li

**Affiliations:** 1 State Key Laboratory of Reliability and Intelligence of Electrical Equipment, Hebei University of Technology, Tianjin 300132, China; huhezhi@hebut.edu.cn; 2 Department of Electronic and Information Engineering, Hebei University of Technology, Langfang 065000, China; 3 Hebei Key Laboratory of Smart Sensing and Human-robot Interactions, School of Mechanical Engineering, Tianjin 300130, China; cjm@hebut.edu.cn (J.C.); 201811201008@stu.hebut.edu.cn (C.W.); 201811201004@stu.hebut.edu.cn (C.Y.); 202021202027@stu.hebut.edu.cn (X.M.); 4 Jiangsu Key Laboratory of Advanced Food Manufacturing Equipment and Technology, Wuxi 214122, China; 5 Institute of Biophysics, School of Science, Hebei University of Technology, Tianjin 300401, China

**Keywords:** microfluidics, digital polymerase chain reaction, degas, meat authentication

## Abstract

Droplet digital polymerase chain reaction (ddPCR) suffers from the need for specific equipment and skilled personnel; thus, we here present a chamber-based digital PCR microfluidic device that is compatible with fluorescence image read-out systems and removes bubbles by a pre-degassed microfluidic device that consists of a pilot channel and micro chamber arrays. Digitalized PCR reagents are introduced into micro chambers, and thermocycles are taken to perform a DNA amplification process. Then, fluorescence images of a micro chamber array are read out and analyzed to obtain the total number of positive chambers. Thereby, the copy numbers of target DNA are calculated for quantitative detections. As a validation, this device is evaluated by the application of meat authentication. We performed dPCR tests using DNA templates extracted from a pure mutton DNA template with different dilutions. Then, the dPCR chip was used to identify the meat authentication using mutton–chicken mixtures with different mass ratios, showing its performance in real biotechnical applications.

## 1. Introduction

The concentration of targets is important in the development of biosensors because in ultra-low concentration situations [1], the background may serve as a substrate noise. Thus, pre-concentration [2,3] or amplification [4] could provide solutions for biosensing. Among these approaches, polymerase chain reaction (PCR) has given access to a method of amplifying target deoxyribonucleic acid (DNA) across several orders of magnitude [5]. Compared with real-time PCR, chamber-based digital polymerase chain reaction (dPCR) or droplet dPCR (ddPCR) enable the absolute quantification [6,7] of nucleic acids without any calibrations. In particular, for microfluidic devices, digitalized micro chambers [8] or droplets [9] are usually used to perform a dPCR or ddPCR. The digitalized micro chamber-based dPCR features micro chamber arrays [10,11] containing a PCR mixture and target DNA for gene amplification, while the droplet-based ddPCR requires a large number (i.e., 20,000 or more) of micro droplets as basic reaction units. Typically, the droplet-based PCR can reach a wider dynamic range due to its advantages in partition numbers. However, it needs external pumps and special devices (i.e., glass capillary [12], co-flow [13] or flow focusing [14] microfluidic chip, even step emulsion microfluidic chip [15], etc.) to generate emulsion droplets, and this in turn causes relatively high costs and the need for specially trained skills. To address this problem, micro chamber-based microfluidic devices have been demonstrated to simplify this process. However, there is still a major problem for chamber-based dPCR, that is, the development of bubbles [16], which could pose problems during read out by fluorescence image measurements. For digital PCR devices with bubble problems, advanced image processing algorithms should be used to identify the bubbles or drops.

The above-mentioned disadvantages highlight the need for an easy-to-operate microfluidic PCR device that could reliably remove bubbles and is compatible with fluorescence image read-out systems. To address this problem, previous studies give a solution from the permeability of PDMS. That is, the high gas solubility of pre-degassed PDMS is exploited to provide the power of self-priming, so that the samples are automatically sucked into the device. Meanwhile, with the negative pressure caused by pre-degassed PDMS device, all the microchambers could be quickly filled with the solution without any air bubbles [17].

Here, we present a 40 × 40 chamber-based digital PCR microfluidic device that is compatible with fluorescence image read-out systems and removes bubbles by a pre-degassing operation. This microfluidic device consists of a pilot channel and micro chamber arrays. Using this device, digitalized PCR reagents are introduced into micro chambers by negative pressures provided by a pre-degassed PDMS layer. Then, 40 PCR thermocycles are performed to complete a DNA amplification process. After that, the fluorescence images of micro chamber arrays are read out and analyzed to obtain the number of total positive chambers. Thereby, the copy numbers of target DNA could be calculated for quantitative analysis.

As a validation, this device and the easy-to-operate method are evaluated by the application of meat authentication. For example, mutton is popular in China and Muslim countries, but it is frequently adulterated with chicken, which is much cheaper [18]. Therefore, the identification of chicken ingredients from mutton using an easy-to-operate microfluidic device is essential for applications. Our previous work has shown the quantitative determination of mutton adulteration using a real-time PCR method [18]; here, we performed dPCR tests for meat adulteration using mutton–chicken mixtures. Firstly, we used DNA templates extracted from pure mutton to get DNA templates and conducted dPCR tests using DNA templates with different dilutions. The main purpose is to evaluate the quantitative performance of our pre-degassed microfluidic chamber-based digital PCR device. Then, the dPCR microfluidic chip was used to identify the meat authentication using meat mixtures with different mutton–chicken mass ratios. We envision that this work will lay the basis for the development of easy-to-operate digital PCR systems that can be employed in biotechnology-related applications.

## 2. Materials and Methods

### 2.1. Principles, Design, and Fabrications of Digital Polymerase Chain Reaction (dPCR) Microfluidic Chip

**Figure 1:**
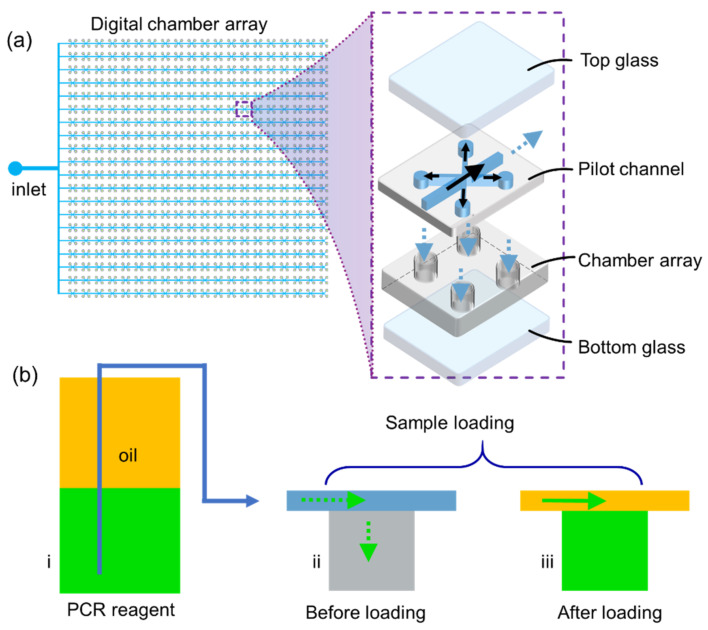
Schematic of chamber array-based dPCR microfluidic chip. (**a**) Left: top view of the chip; right: exploded view of one basic unit of the dPCR microfluidic chip. (**b**) Principles of sample loading.

A schematic illustration of the glass–PDMS (polydimethylsiloxane)–glass dPCR microfluidic chip is shown in Figure 1. As shown in Figure 1a, from the top view of the chip, it consists of one inlet and 40 × 40 digital chambers (150 μm in diameter, 30 μm in height). As demonstrated in the exploded view of one basic unit of the chip, it consists of four layers. From the top to bottom, there is a top glass layer for sealing purposes, a pilot channel layer to introduce the PCR reagent mixtures (also oil phase) into the digital chambers, which are below the pilot channel layer, a micro chamber array layer to hold the PCR reagents and take DNA amplification reactions, and a bottom glass layer as a substrate. In fact, the chip has three layers (top glass, middle PDMS, and bottom glass) physically; the pilot channel and the chamber array are within the same PDMS layer fabricated by a two-layer SU-8 (Microchem, Westborough, MA, USA) mold. Here, in the exploded view of one basic unit of the dPCR microfluidic chip, the four-layer structure is just an illustration to show the functions of pilot channel and micro chambers.

To fabricate the PDMS layer shown in Figure 1a, a SU-8 mold for the pilot channel and micro chambers was firstly fabricated by two-layer lithography technology [19]. Then, PDMS base and curing agents (Sylgard 184 Silicone elastomer kit, Dow Corning, Midland, MI, USA) were well mixed (10:1% w/w) and degassed to remove air bubbles. Next, the PDMS prepolymer was cast on the two-layer SU-8 mold and cured for 3 h at 60 °C or overnight at room temperature. Then, the inlet was punched in the PDMS top layer with a 1 mm biopsy puncher (Suzhou Wenhao Co. Ltd., Suzhou, China).

Once the PDMS layer was ready, the bottom glass layer was plasma bounded with the PDMS layer for 60 s at 100 W (Henniker HPT-200, Runcorn, UK). To note, the size of the bottom glass should no smaller than the size of the PDMS layer. However, the top glass layer should be smaller than the PDMS layer in order to leave the inlet area out. The purpose of this design is to load PCR reagents or oil samples into the micro chambers without external actuators. After the PDMS and bottom glass were plasma bounded, the chip was degassed (−1 kPa, 30 min) in a vacuum box (Fujiwara PC-3, Taizhou, China) to get a pre-degassed microfluidic chip. Due to the air permittivity of the PDMS block [20,21], the degassed chip was compressed and was able to provide negative pressures, which provide the power to pump any reagent from the inlets. Thus, the dPCR microfluidic chip is an external power-free device. The illustration of sample loading is shown in Figure S1 in the Supplementary Materials.

The principles of a degassed power free microfluidic chip are demonstrated in Figure 1b. To load the PCR samples into all the micro chambers, there are three know-hows to operate our device in the sample loading process: (1) The volume of the PCR reagents was determined by the internal volume of digital chamber arrays. (2) An oil phase was added on the top of the PCR reagent mixtures, which are usually stored in a centrifuge tube. (3) Before loading the samples into the inlet, a glass cover is put onto the top surface of the PDMS layer. The reasons are discussed in detail in Section 4.

### 2.2. dPCR Evaluation and Meat Authentication Tests 

Meat samples (mutton and chicken) were purchased from the local supermarket. Seven kinds of reference meat samples including two control samples and five experimental samples were prepared, as listed in Table 1. All the meat samples were stored in a -20℃ refrigerator before tests. A commercial Genome DNA extract kit was used from meat samples. The mutton-specific primers sequences (5′ 3′) and probe are CTGACACACGGGACACMTCTCC (Forward), AAGCTAAACATGGACCCACAT (Reverse), and FAM-TAAGCCAGCCTT-GTGCGTGTGGTGTGGTCC-BHQ1 (Probe). The chicken-specific primers and probe are AGCAATTCCCTACATTGGACACA (Forward), GATGATAGTAATACCTGCGATTGCA (Reverse), and HEX-ACAACCCAACCCTTACCCGATTCTTC-BHQ1′. They were synthesized by Genewiz Inc. (South Plainfield, NJ, USA).

A commercial PCR kit set (KAPA kit, Sigma-Aldrich, St. Louis, MO, USA) was used to evaluate the performance of our dPCR microfluidic chip. The PCR amplification was taken by an initial heat at 95 °C for 3 min denaturation, followed by 40 cycles (95 °C for 30 s and 60 °C for 32 s) and a finally heat at 72 °C for 2 min. The heating/cooling process was carried out on the microfluidic chip using a self-developed TEC (thermoelectric coolers) controller.

As a demonstration, we firstly performed an evaluation of the micro chamber array-based digital PCR microfluidic chip using DNA templates extracted from control sample 2 (pure mutton). To verify its quantitative performance, the DNA templates were pre-processed with different dilutions. Then, the dPCR chip was used to identify the meat authentication using mutton and chicken, showing its performance in real biotechnical applications.

### 2.3. Data Acquisition and Analysis

The fluorescence images of a micro chamber array dPCR chip were observed by the use of an inversed fluorescence microscope (Nikon Eclipse Ti-S, Tokyo, Japan) equipped with a CCD ( Charge-coupled Device ) camera (Nikon DS-Qi2, Tokyo, Japan) to record fluorescence images after PCR cycles. The recorded images of each sample were further analyzed using ImageJ software. 

To qualitatively assess the performance of our dPCR microfluidic chip, post-processing of fluorescence images was obtained by setting up a threshold value to identify the positive chambers from negative ones. The number of positive chambers may vary with threshold values; thus, it is important to find a suitable threshold value to identify positive chambers from negative ones. Since Gaussian distribution (normal distribution) is the most common distribution function for independent, randomly generated variables, so we used Gaussian distribution to describe the fluorescence intensities or gray scales. The threshold is expressed as

(1) $$I_{\mathrm{thr}}=\mathrm{MAX}\left\{\mu_{1}-3\sigma_{1},\mu_{2}-3\sigma_{2},\;\ldots \right\}$$

where MAX{ } stands for returning the largest value in a given list of arguments. $\mu_{i}$ and $\sigma_{i}$ (i = 1, 2) are the mean and standard deviation of Gaussian distribution, respectively. The distribution of the fluorescence intensities or gray scales may have one or two peaks; thus, we defined both situations for unimodal distribution or bimodal distribution. If all the fluorescence intensities or gray scales show unimodal distribution, then the threshold would be μ−3σ. Otherwise, if there is more than one peak, the threshold to distinguish positive from negative should be defined by Equation (1).

The number of positive chambers from the fluorescence images were read out and then evaluated using Poisson distribution equation, as the following [22]

(2) $$N_{\mathrm{cal}}=\frac{\lambda }{v}=\frac{-\ln\left(1-N_{\mathrm{read}}/n\right)}{\pi D^{2}h/4}$$

where $N_{\mathrm{cal}}$ stands for the calculated copy numbers of target DNA; $N_{\mathrm{read}}$ is the read out the number of positive chambers from the experimental fluorescence images; n is the total number of micro chambers; and D,h stands for the diameter and height of the micro chamber.

## 3. Results

### 3.1. Evaluation of dPCR Microfluidic Chip

**Figure 2:**
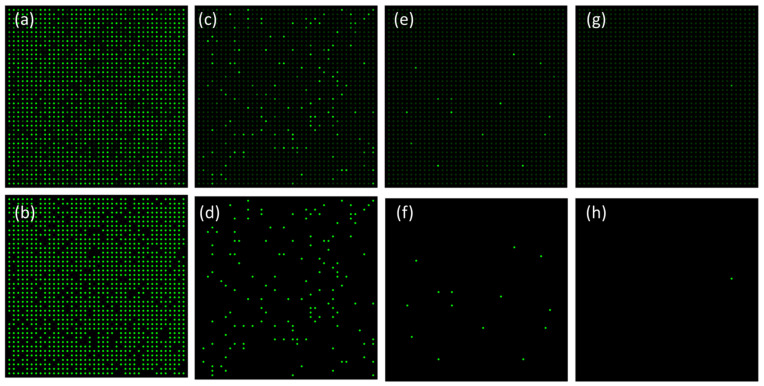
Fluorescence images before and after image process to read out the total number of positive chambers. (**a**,**c**,**e**,**g**) are original images from the CCD camera, with DNA template concentration of 1 × fold, 0.1 × fold, 0.01 × fold, 0.001 × fold dilutions, respectively; (**b**,**d**,**f**,**h**) are post-processed images accordingly.

As shown in Figure 2, we demonstrated the fluorescence images before and after the image process to read out the total number of positive chambers. From evaluations with an ultraviolet spectrophotometer, the initial concentration of DNA template is 3912 copies/μL. Figure 2a,c,e, and g are original images from the CCD camera, with DNA template concentrations of 1 × fold, 0.1 × fold, 0.01 × fold, and 0.001 × fold dilutions, respectively. Figure 2b,d,f and h show their post-processed images accordingly.

Take Figure 2a and b for example; with an initial concentration without any dilutions, the number of positive chambers could be up to 1344, which is 84% of the overall micro chambers. In Figure 2b, all the positive chambers are highlighted by strong fluorescence intensity, while the negative chambers are displayed as dark chambers.

**Figure 3:**
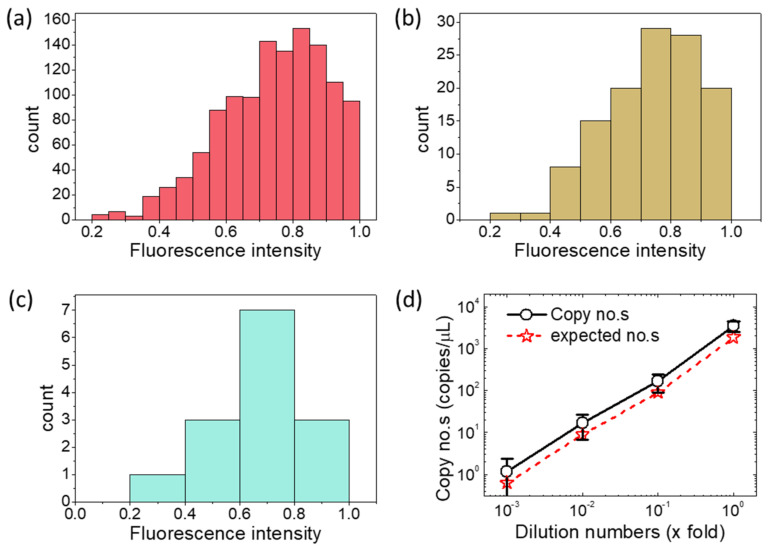
Evaluation of the dPCR microfluidic chip by quantitative analysis. (**a**–**c**) are histogram plots of original images from the CCD camera, with DNA template concentration of 1 × fold, 0.1 × fold, 0.01 × fold dilutions, respectively. (**d**) is the comparison of expected copy numbers and calculated copy numbers from three independent tests.

The fluorescence image series in Figure 2 suggests that at lower DNA template concentrations, a significant decrease in the number of positive chamber is observed. The number of positive chambers in Figure 2c,e,g are 136 (namely 8.5% of the overall chambers), 14 (namely 0.875% of the overall chambers), and 1 (namely 0.0625% of the overall chambers), respectively. This is also confirmed by the statistical analysis histogram, as shown in Figure 3.

It can be seen from Figure 3 that the positive chamber count has a norm distribution with a mean value around 0.7–0.8. Here, the x-axis in Figure 3a–c stands for the normalized fluorescence intensity, which is defined by *I*_ij_ /*I*_max_ (here, *I*_ij_ is the fluorescence intensity for the chamber at line i and row j; and I_max_ is the maximum value of fluorescence intensities among all the 40 × 40 chambers). From Figure 3d, the calculated DNA copy numbers at each dilution show a linear relationship with the expected copy numbers in logarithmic coordinate system. In addition, it was found that the calculated target DNA copy numbers are slightly bigger than the expected copy numbers.

### 3.2. Applications in Meat Authentication

After the evaluations of our dPCR microfluidic chip using a standard PCR kit set, we applied this device to meat authentication experiments. The influence of chicken/mutton mass ratios was investigated by placing DNA templates from seven meat samples (includes two control samples and five experimental samples, as listed in Table 1) into our dPCR microfluidic chip and running PCR cycles. We put the images before and after post-processing, as shown in Figure S2 in the Supplementary Materials.

**Figure 4:**
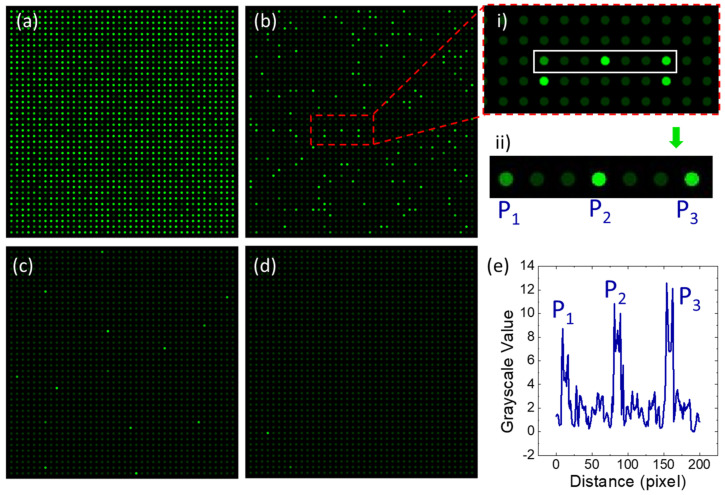
Applications in meat authentication for mixtures of mutton and chicken samples. (**a**–**d**) are fluorescence images read by the CCD camera, for experimental samples 1–4 listed in Table 1, with chicken/mutton mass ratio of 1:1, 1:10, 1:100, and 1:1000, respectively. (**e**) is the plot of gray scale as a function of distance for the part of images shown in subfigure (**b**).

Figure 4a–c, and d show the fluorescence images recorded by a CCD camera. Each image is from meat samples with different chicken/mutton mass ratios. Hereby, the images of two control samples (pure chicken and pure mutton) and one experimental sample (meat mixture with chicken/mutton mass ratio of 1:10,000) were not shown because nearly 100% or 0% positive chambers were observed for these conditions.

It can be seen from Figure 4a–d that the count of positive chambers is decreasing with the decreasing chicken/mutton mass ratios. Take Figure 4a–d for example, the chicken/mutton mass ratio is ten times as diluted as 1:1, 1:10, 1:100, and 1:1000, respectively. Accordingly, the read-out numbers of positive chambers with the above dilutions are 1496, 148, 14, and 2, respectively. From Equation (2), the calculated copy numbers of these dilutions are 2240 copies/μL, 79.2 copies/μL, 7.2 copies/μL, and 1.0 copies/μL.

To show the comparation of positive and negative chambers, a small area of Figure 4b was selected to analyze the gray scale as a function of distance, as shown in Figure 4e. The area highlighted with a rectangle in Figure 4b(i,ii) shows the location of target chambers analyzed in Figure 4e. The gray-scale value of positive chambers is above 8, while the gray-scale value of negative chambers is only around 2, showing a significant difference between positive and negative chambers.

## 4. Discussion

### 4.1. Impact of Sample Loading on the Quantitative Performance

As mentioned in Section 2, the micro chamber array-based digital PCR microfluidic chip was designed to take 40 × 40 independent DNA amplification tests for one single run. Each chamber features an individual PCR test by their fluorescence intensities. From the fluorescence intensities of each chamber, the total copy numbers of DNA template could be calculated by Equation (2).

As shown in Figure 3d, the calculated DNA copy numbers (of pure mutton sample) at each dilution show a linear relationship with the expected copy numbers in a logarithmic coordinate system. However, the calculated target DNA copy numbers are slightly bigger than the expected copy numbers at each dilution condition. The most likely reason for this phenomenon may rely on the errors from sample loading.

Figure 1b demonstrated the principle of the sample loading process. We did not punch any outlets in the dPCR microfluidic chip, and also, the flow and digitalization of PCR reagents are achieved from negative air pressures caused by the pre-degassed PDMS layer. Therefore, the sample loading process is highly nonlinear; thus, the flow rate or total volume of fluids could not be controlled at a high precision level. From this point, the total volume of PCR reagents might influence the quantitative performance of our dPCR microfluidic chip. To enhance the degassing efficiency, we did not put the top glass cover before degassing. However, when loading the samples into the inlet, a glass cover should be placed onto the top surface of the PDMS layer to prevent unnecessary air leakages.

**Figure 5:**
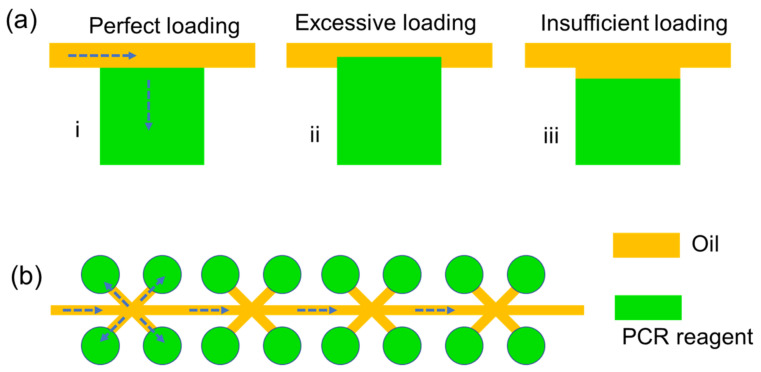
Schematic of chamber array-based dPCR microfluidic chip. (**a**) Cross-section view of two-layer lithography fabricated PDMS layer, including a pilot channel filled with oil, and a group of micro chambers filled with PCR reagents; left: the case of perfect sample loading; middle: the case of excessive sample loading; right: the case of insufficient sample loading. (**b**) Top view of two-layer lithography fabricated PDMS channel or single-layer lithography fabricated PDMS channel.

Ideally, the expected PCR reagent volume needs to be exactly equal to the volume of a single micro chamber, as shown in Figure 5a(i). In this case of perfect sample loading, all the PCR mixture reagents would occupy the 1600 micro chambers within the chip. Meanwhile, the oil phase would occupy the pilot channels.

As shown in Figure 1b and Figure 5, there is an oil phase upon the PCR reagents, no matter before or after sample loadings. The most important reason to introduce oil phase into the dPCR microfluidic chip is that it could work as the seal layer to avoid any evaporations during PCR heating reactions. Our previous tests showed that if we did not introduce the oil phase as we did in this work, the volume of PCR reagents would decrease with time, leading to a significant error in the calculated copy numbers. However, on the other hand, the introduction of the oil phase also might impact the precisions of sample loading. For example, as shown in Figure 5a(ii), in the case of excessive loading situations, the volume of the PCR mixture reagents in the experiments was bigger than the internal volume of digital chamber arrays; thus, the real volume per digital drop would be bigger than expected (volume of one single micro chamber). As a result, the calculated copy numbers obtained from Equation (2) would be smaller than the expected copy numbers. On the contrary, as demonstrated in Figure 5a(iii), the calculated copy numbers would be bigger than expected in the case of insufficient loading conditions. This is reasonable because excessive or insufficient loading may occur if the pipette is not well calibrated.

The schematic shown in Figure 5b provides a top view for the PDMS channel, no matter whether the SU-8 mold is fabricated by two-layer or single-layer lithography. Compared with single-layer lithography fabricated PDMS channel-chamber devices, the two-layer lithography fabricated device shows better sample loading performance in our tests. Thus, we used a two-layer lithography technology to fabricate the SU-8 mold for the PDMS layer, which consists of a pilot channel and 40 × 40 digitalized micro chambers. This design is similar but different from the vertical branching microchannel microfluidic chip proposed by Si et al. [10]. In Si’s work, a glass–PDMS–glass “sandwich” structure was employed in the chip to form a robust support. Moreover, the main channel layer of their vertical branching microfluidic chip is under the digital micro chambers, while in our design, the pilot channel is at the top of micro chamber arrays. The aim of this design is to obtain a better sample loading performance. Although the gravity of fluids at the micro scale is much smaller than viscous forces, the gravity of PCR reagents that is expected to fill the micro chambers is negligible, but the step structure could prevent backflows, which is observed if the pilot channels and micro chamber are fabricated with single-layer lithography.

The parallel array adopted in this work also could achieve a fully self-digitalization within minutes, as the branching array. Moreover, the sample loading time could even be as short as tens of seconds, depending on the negative pressure and degassing time before sample loadings. With the conditions of −1 kPa and 30 min degassing, as mentioned in Section 2.1, the PCR reagents could fully fill all the 40 × 40 chambers within one minute. If we increase the negative pressures or degassing time (i.e., −2 kPa, 40 min), the sample loading could be finished in about 40 s.

### 4.2. Performance for Meat Authentication Analysis

DNA templates extracted from control sample 2 (pure mutton) with different dilutions were used to evaluate the quantitative performance of our dPCR microfluidic chip. Following similar procedures, its application in meat authentication analysis was also employed in this work.

The fluorescence images shown in Figure 4a represent the distribution of positive and negative chambers when the meat sample with chicken/mutton mass ratio of 1:1 was used to extract DNA template. Due to the specificity of probes for chicken meat, most of the micro chambers show positive, indicating that there are chicken contents in experimental meat sample 1, as listed in Table 1. In order to investigate the lower limit of detection of this method, more meat samples (samples 2–5, as listed in Table 1) with lower percentage of chicken contents were used to validate the performance of this device in the application of meat authentication analysis. Once we decrease the mass percentage of chicken (chicken mass percentage: 1/2, 1/11, 1/101, and 1/1001, respectively), it was observed that the chicken-positive chamber counts also decrease, as shown in Figure 4b–d. These results demonstrate that the dPCR method is sensitive and specific for the rapid identification of chicken meat mixed in mutton.

Since the number of target DNA copies varies with different cells and tissues [18], it is reasonable to lead quantification errors for meat authentication. In Figure 4d, as the experimental result from a single test, we observed two positive chambers, but there were not always two positive chambers for the dPCR tests of meat sample 4. Actually, it was frequently observed that there are no positive dots being observed from the CCD camera. Furthermore, as the chicken percentage goes down as low as 1/10,001 (namely for meat sample 5 in Table 1), it is more likely to see zero positive chambers. Thus, we propose that the lower limit of detection might be a chicken/mutton mass ratio of 1:100 or 1:1000.

For economic profit, the inclusion of poultry products in other meats is over 10% of the total meat weight [23]; thus, the dPCR microfluidic chip is still available for the rapid detection of meat authentication applications.

## 5. Conclusions

We presented an easy-to-operate chamber-based dPCR device with a pre-degassed microfluidic chip consisting of a pilot main channel and 40 × 40 micro chamber arrays. With this device, PCR reagents could be self-digitalized. As a demonstration, a mutton–chicken mixture was used to show its performance in meat authentication. After a thermocycling performed on the chip, fluorescence images of micro chamber array are read out and analyzed to calculate copy numbers of target DNA. Compared to existing chamber-based dPCR solutions, this device offers an easier sample loading performance. Although the lower limit of detection (1:100 to 1:1000) cannot get the best performance at the current stage, it is still available for the rapid detection of meat authentication applications.

## Figures and Tables

**Table 1:** Reference meat samples used in this work.

Category	Chicken Mass	Mutton Mass	Mass Ratio
control 1	10 g	/ ^1^	/
control 2	/	10 g	/
sample 1	10 g	10 g	1:1
sample 2	10 g	100 g	1:10
sample 3	1 g	100 g	1:100
sample 4	1 g	1000 g	1:1000
sample 5	100 mg	1000 g	1:10000

^1^ Stands for no mass or non-mathematical significance.

## Supplementary Materials

The following are available online at https://www.mdpi.com/article/10.3390/mi12060694/s1, Figure S1: Schematic of the sample loading process with a cross-section view. Figure S2: Experimental results of meat authentication applications with the fluorescence images before and after image process to read out the total numbers of positive chamber.
