# Supplementary material for: Pre-Degassed Microfluidic Chamber-Based Digital PCR Device for Meat Authentication Applications
Source: Micromachines (Basel). 2021 Jun 14;12(6):694. doi: 10.3390/mi12060694 (PMC8231815; doi:10.3390/mi12060694)
Supplement: Supplementary file 1 [file micromachines-12-00694-s001.zip › micromachines-1251850-supplementary.pdf]

micromachines

Supplementary materials

Pre-Degassed Microfluidic Chamber-Based Digital PCR Device for Meat Authentication Applications

Hezhi Hu [1,2,†], Jingmeng Cheng [3,†], Chunyang Wei [3], Shanshan Li [1,3,4,*], Chengzhuang Yu [3], Xiaoshuai Meng [3] and Junwei Li [2,5,*]

1 State Key Laboratory of Reliability and Intelligence of Electrical Equipment, Hebei University of Technology, Tianjin 300132, China; huhezhi@hebut.edu.cn
2 Department of Electronic and Information Engineering, Hebei University of Technology, Langfang 065000, China
3 Hebei Key Laboratory of Smart Sensing and Human-robot Interactions, School of Mechanical Engineering, Tianjin 300130, China; cjm@hebut.edu.cn (J.C.); 201811201008@stu.hebut.edu.cn (C.W.); 201811201004@stu.hebut.edu.cn (C.Y.); 202021202027@stu.hebut.edu.cn (X.M.)
4 Jiangsu Key Laboratory of Advanced Food Manufacturing Equipment and Technology, Wuxi 214122, China
5 Institute of Biophysics, School of Science, Hebei University of Technology, Tianjin 300401, China
* Correspondence: sli_mems@hebut.edu.cn (S.L.); junwei_li@hebut.edu.cn (J.L.); Tel.: +86-22-60202605 (S.L.); Tel.: +86-22-60201070 (J.L.)
† These authors contribute equally.

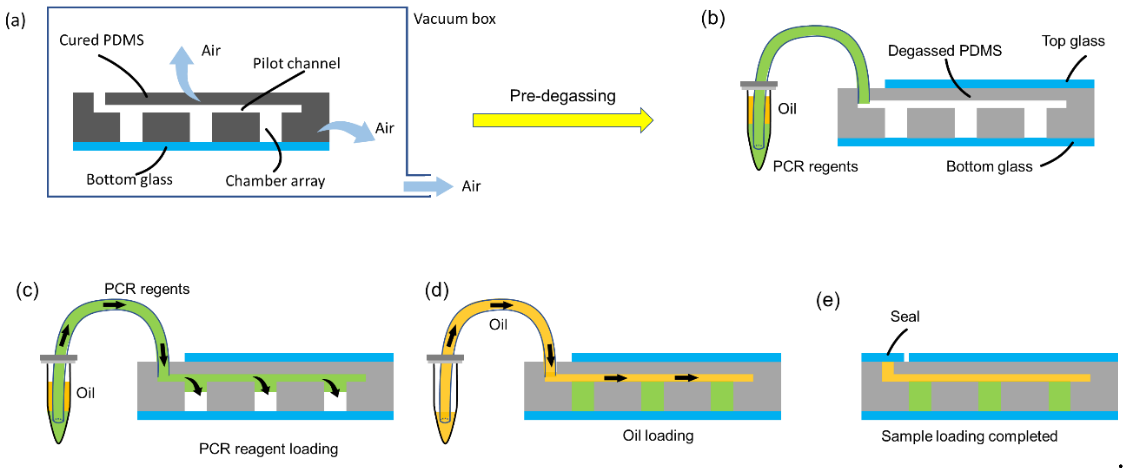

Figure S1. Schematic of the sample loading process with a cross-section view. (**a**) The pre-degassing in a vacuum box. (**b**) Pre-degassed dPCR chip. (**c**) PCR reagent loading. (**d**) Oil loading. (**e**) Sample loading completed.

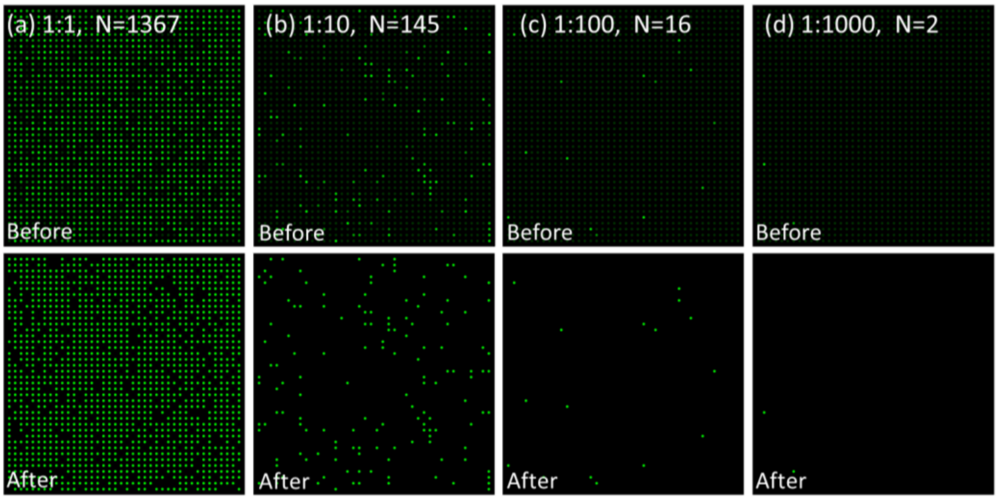

Figure S2. Experimental results of meat authentication applications with the fluorescence images before and after image process to read out the total numbers of positive chamber. From (**a**) to (**d**), the experimental meat samples are with chicken/mutton mass ratio of 1:1, 1:10, 1:100, and 1:1000, respectively.
